# Variability in the Responsiveness to Low-Dose Aspirin: Pharmacological and Disease-Related Mechanisms

**DOI:** 10.1155/2012/376721

**Published:** 2012-01-11

**Authors:** Bianca Rocca, Giovanna Petrucci

**Affiliations:** Department of Pharmacology, Catholic University School of Medicine, 00168 Rome, Italy

## Abstract

The main pharmacological aspects of pharmacodynamics (PD) and pharmacokinetics (PK) of aspirin as antiplatelet agent were unravelled between the late sixties and the eighties, and low-dose aspirin given once daily has been shown to be a mainstay in the current treatment and prevention of cardiovascular disorders. Nevertheless, several PD and PK aspects of aspirin in selected clinical conditions have recently emerged and deserve future clinical attention. In 1994, the term “aspirin resistance” was used for the first time, but, until now, no consensus exists on definition, standardized assay, underlying mechanisms, clinical impact, and possible efficacy of alternative therapeutic interventions. At variance with an undefined aspirin-resistant status, in the last 5 years, the concept of variability in response to aspirin due to specific pathophysiological mechanisms and based on PK and/or PD of the drug has emerged. This growing evidence highlights the existence and possible clinical relevance of an interindividual variability of pharmacological aspirin response and calls for new, large studies to test new low-dose aspirin-based regimens which may ameliorate platelet acetylation, reduce variability in drug responsiveness, and improve clinical efficacy on selected populations.

## 1. Introduction

In 1982, the Nobel Prize in Physiology or Medicine was awarded jointly to Sune K. Bergström, Bengt I. Samuelsson, and John R. Vane for their discoveries during the sixties and early seventies of prostaglandins and related biologically active substances. They also showed that aspirin and aspirin-like drugs inhibited prostaglandin biosynthesis from arachidonic acid (AA) and that this was the basis for their therapeutic anti-inflammatory, antipyretic, and analgesic effects [1, 2]. The initial descriptions of a platelet-inhibiting effect of aspirin in the late sixties were based on assays of hemostasis and platelet function available at that time, such as the bleeding time and ADP-induced optical aggregation [3, 4]. On the basis of those assays, aspirin was described as a weak antiplatelet agent, causing a “mild prolongation of the bleeding time” and a “minor hemostatic defect” in normal subjects [3–5]. Few years later, Smith and Willis demonstrated that aspirin was able to block prostaglandin production from human platelets [6], and the group of Samuelsson identified thromboxane (TX) A_2_ as the biologically active prostanoid synthesized from AA in activated platelets and blocked by aspirin [7].

 In the mid seventies, P. Majerus and collaborators unravelled the mechanism of action of aspirin at the molecular level. Using proteins purified from human platelets and aspirin radiolabelled in the acetyl residue (^3^H-acetyl aspirin), they showed that aspirin rapidly (within minutes) and irreversibly acetylated a specific protein fraction of approx. 85 kDa within the AA-binding, active site, and this protein corresponded to human cyclooxygenase (COX) [5, 8, 9]. Aspirin acetylated the 85 kDa platelet's fraction in a saturable manner and at concentrations relatively lower (up to 30 *μ*M) than the ones required to acetylate other purified proteins such as albumin, immunoglobulins, or fibrinogen [5]. Moreover, different groups reported that COX in intact platelets was acetylated or inhibited *in vitro* by aspirin concentrations lower than the ones required in other nucleated cellular systems (human synovial tissue, smooth muscle cells, fibroblasts, and sheep seminal vesicles) [5, 9, 10], indicating a possible, cell-milieu-dependent modulation of the enzymatic COX activity. Approximately twenty years later, P. Loll and coworkers resolved the X-ray crystal structure of COX-1 bound to aspirin [11].

**Figure 1:**
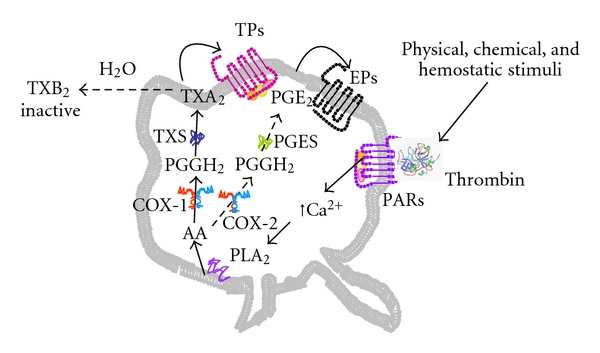
Cyclooxygenase-dependent arachidonic acid pathway in platelets. Thrombin, generated *in vivo* or *ex vivo* by several chemical or physical stimuli, activates its protease-activated receptors (PARs) increasing intraplatelet calcium, which triggers phospholipases (PL) A_2_-dependent cleavage of arachidonic acid (AA) from plasma membranes. AA is the enzymatic substrate of cyclooxygenase (COX)-1 and -2. COX-1-dependent AA path in platelets generates mainly TXA_2_ which amplifies platelet activation by binding to its platelet receptors (TPs). COX-2-dependent AA path in normal platelets is less prominent and generates mainly PGE_2_ which acts as a positive modulator of platelet response to other agonists by binding to its platelet receptors (EPs). TXA_2_ both *in vivo* or *ex vivo* is nonenzymatically hydrolized to TXB_2_, which is biologically inactive but stable, and can be measured in *ex vivo* assays or undergoes further hepatic enzymatic biotransformation *in vivo*.

 The pharmacokinetics (PK) of oral aspirin in healthy volunteers, especially in a wide dose range, including low doses (between 25 and 160 mg/day), was described soon thereafter by different groups in Europe and in the United States [8, 12–15]. The description of aspirin PK was very much facilitated by an *ex vivo* method introduced by C. Patrono and collaborators, reflecting the entire enzymatic COX-dependent activity of platelets in the peripheral blood [13]. In fact, until that time, methods for studying aspirin inhibition *ex vivo* in humans were quite laborious and used an *in vitro* mixing of ^3^H-acetyl aspirin and blood from aspirin-treated subjects [8, 9], or on TXB_2_ measured in aggregated platelet-rich plasma [14]. These methods required relatively large amounts of blood, platelet isolation, extraction of protein fractions, or aggregation reactions and were time consuming and scarcely applicable to large-scale studies. Moreover, the method with ^3^H-acetyl aspirin explored the degree of acetylation of platelet's COX protein by aspirin, but it did not measure the level of inhibition of COX enzymatic activity leading to TXA_2_ generation. The method described and validated by Patrono and collaborators required minimal blood volume and little preanalytical handling and was relatively rapid. It was based on a physiological hemostatic reaction: during whole blood clotting at 37°C, endogenous thrombin is physiologically generated. Thrombin is one of the strongest trigger of platelet's AA release [16], maximally fuelling the enzymatic activity of COX and the subsequent biosynthesis of TXA_2_ in platelets (Figure 1). TXA_2_ is extremely labile in an aqueous milieu and is nonenzymatically hydrolyzed to TXB_2_, which is a stable derivative and measurable in serum by immunometric assays without purification steps [13]. Thus, this biochemical method closely reflects the maximal biosynthetic capacity (and its degree of inhibition) of platelet's COX enzyme in a physiological environment, such as whole blood and endogenous thrombin.

The pharmacology of aspirin in humans, especially in the low-dose range, was described by those methods, and its main characteristics can be summarized as follows. The effect of aspirin repeatedly administered once daily is irreversible, cumulative, saturable, reaching a ceiling effect in the low-dose range, for instance, at approximately 100 mg of plain aspirin in single dose or 20–40 mg for repeated (approx 10 days) daily dosings [12, 13]. Aspirin acetylates platelet's COX-1 already in the presystemic, portal blood, before the liver first pass [15]. Finally, the time course of a nearly complete platelet cyclooxygenase inhibition by repeated low doses, and conversely, the time course of the recovery of platelet COX activity after aspirin withdrawal takes approximately 7–10 days, reflecting two characteristics of platelets: their almost-complete inability to replace the acetylated enzyme and their lifespan [9, 12–14]. Moreover, following aspirin withdrawal, even after lower doses, different studies showed a two-day lag before the appearance of a significant new, nonacetylated COX protein or COX enzymatic activity in circulating platelets [8, 13]. This delay was observed independently of the techniques used (radiolabelled aspirin and serum TXB_2_), and it likely reflects the acetylation of megakaryocytes, which, at least in conditions of normal megakaryopoiesis, during the first 24–48 hour after aspirin withdrawal, largely release in the peripheral blood platelets with acetylated, nonfunctioning COX enzymes [8, 13]. These data were confirmed in a more recent study [17]. This lag interval has also been reported in different mammalian species [18, 19], compatibly with the species-specific kinetics of megakaryopoiesis.

 Following the description of low-dose aspirin PK, the clinical benefits of 160 mg once daily (enteric coated formulation) was tested for the first time on a large number of acute myocardial infarction (MI) patients in the ISIS-2 trial [20]. In that trial, aspirin reduced by approximately 25% the vascular death in acute MI patients as compared to placebo. A meta-analysis of the antiplatelet's trialist's collaboration of clinical trials on high-risk populations showed that the cardiovascular protection of aspirin is “saturable” at daily doses of aspirin between 75 and 160 mg day [21], similarly to the dose range which reaches the ceiling effect in inhibiting serum TXB_2_ [13]. Daily doses beyond 160–325 mg day do not add clinical benefit, versus placebo, while increase the bleeding risk and are associated with a trend in a reduction of cardiovascular protection, presumably reflecting the inhibition of prostacyclin in the vessel wall [21, 22].

## 2. From Aspirin Resistance to Variability in Responsiveness

**Figure 2:**
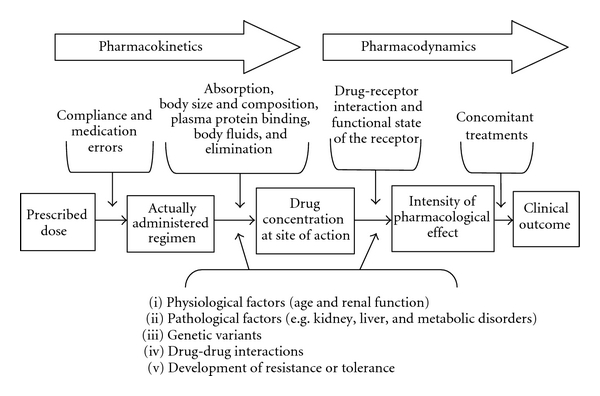
Pharmacokinetics, pharmacodynamics and pathophysiological conditions affecting the clinical outcome of a drug. The figure depicts the different pharmacokinetics and pharmacodynamics steps possibly involved in drug transformation and clinical effect. Pathophysiological conditions which may affect both PK and/or PD are also reported in the figure. This figure is modified from [24].

Practicing physicians have long recognized that individual patients show wide variability in response to the same drug or treatment. The concept of interindividual variability in the response to any drug can be exemplified as follows: a range of drug plasma concentrations is often required to produce an effect of a specified intensity in all the patients; on the other hand, at a specified plasma drug concentration, an effect of varying intensity will occur in different individuals. Drug's PK encompasses drug absorption, distribution, metabolism (biotransformation), and elimination, thus contributing to the quite variable plasma concentrations of the drug and/or its active metabolite(s) in different individuals receiving the same dose. Pharmacodynamics (PD) refers to the biochemical and physiological effects of drugs and their mechanisms of action, which result in a complex relationship between a given concentration and the magnitude of the observed clinical response [23]. Drug's PK and PD encompass the majority of sources of inter- and intraindividual variability in drug response (Figure 2) and are the basis to understand any treatment success, failure, or adverse reactions. Recommended dosage regimens are usually designed for an “average” patient, but adjustments may be required by specific diseases or physiological factors.

Drug resistance, a well-characterized phenomenon in the field of chemotherapy, is only one possible cause of a modified (variable) response to a drug [23]. Usually, drug resistance pertains to the PD of a drug (direct or indirect changes in drug-target interaction), is drug-induced, and is stable over time once it has been triggered and obliges to treatment interruption [29]. The main features of variability versus resistance are depicted in Table 1. At variance with any other class of drugs, the characterization of “aspirin resistance”, escaped any well-established pharmacological definition or mechanism [30–32]. Aspirin resistance has been heterogeneously defined on a clinical basis as treatment failure and/or on a functional basis as lower-than-expected responsiveness to different, nonstandardized platelet functional assays [30–32]. However, agreement between different platelet functional assays is less than optimal, percentage of resistance is very much assay dependent, studies on “resistance” are mainly retrospective and not controlled for compliance or NSAID intake [17, 33, 34]. Thus, aspirin “resistance” still lacks consensus on definition, reference assay, pathogenetic mechanisms, and, unlike a true drug resistance, is very often an unstable phenotype over time [17, 30]. An aspirin-induced, time-dependent change in aspirin's target (e.g., COX-1 and/or -2), as shown for antibiotic or antiblastic resistance, identified by a standardized assay and successfully treated with a different antiplatelet agent, has never been reported. Thus, no pharmacological, evidence-based strategies can be rationally applied to understand and treat a “resistant” patient.

 At variance with an undefined “resistance”, as for any other drug, aspirin responsiveness in different patients can be varied by physiological or pathological conditions affecting drug's PK or PD mechanisms. Thus, in 2005, a different concept surfaced on aspirin treatment, for example, the possibility of PK- or PD-based variability in aspirin responsiveness of the individual patient [24, 35, 36].

## 3. Pharmacokinetic and/or Pharmacodynamic Sources of Variability in Aspirin Response

**Figure 3:**
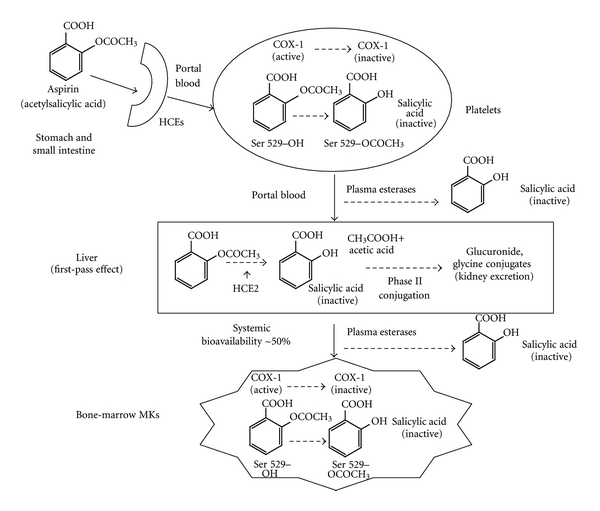
Pharmacokinetics of aspirin. Aspirin is absorbed in the stomach and small intestine, exerts its pharmacodynamic effect, for instance, the acetylation of a Serine (Ser) 529 residue of COX-1 already in the portal blood, and is biotransformed to inactive salicylic acid by intestine, plasma, and liver esterases. On average, its systemic bioavailability is approx. 50% of the administered dose. Once in the systemic circulation, aspirin reaches bone-marrow megakaryocytes (MKs) and inhibits COX-1 and -2 of MKs and developing platelets. HCE: human carboxylesterase.

As compared to other antiaggregants, the PK of aspirin is quite straightforward (Figure 3). Plain aspirin is rapidly absorbed by passive diffusion as undissociated salicylic acid from the stomach, where the pH is low and hydrolysis is minimal, and from the upper small intestine. Oral bioavailability of acetylsalicylic acid is approximately 50% because a fraction of the administered and absorbed dose of the drug is inactivated, that is, deacetylated, by the carboxylesterases in plasma and liver before entering the systemic circulation (*first-pass effect*). The hepatic human carboxylesterase-2 (HCE2) isoform is mainly involved in the first-pass aspirin bioinactivation as compared to the HCE1 isoform [37, 38]. Inactivation may also occur in the gut by means of the same esterases. Furthermore, aspirin can be hydrolized in the peripheral blood by some plasma cholinesterases [39], erythrocyte arylesterases [40], and other esterases called “aspirin esterases” [39]. After a single oral dose of plain aspirin, plasma peak is reached in approximately 1 hour, its plasma *t*1/2 is 20 minutes [41]. The PD of aspirin pertains its interaction with and blockade of the active site of COX-1 and/or -2, being aspirin a nonselective inhibitor of both COX-1 and -2 isoforms. The main site of platelet's COX acetylation in the low-dose range of aspirin is portal blood, before the first-pass effect [15].

Aside from compliance, which is a critical issue especially in chronic patients [42], possible sources of variability in aspirin responsiveness due to modification of its PK and/or PD might be aspirin formulations, body size, ageing, drug-drug interaction, and rate of the drug target turnover, for example, platelet's COX-1 and -2.

 Enteric-coated (EC) aspirin formulations, now widely used in cardiovascular disease prevention and treatment, have been conceived on the hypothesis of resisting the disintegration of the pill in the acid environment of the stomach, thus releasing the drug into the upper small intestine, and avoiding a local damaging effect of acetylsalicylic acid in the stomach [43]. However, evidence supporting a better gastrointestinal safety of EC aspirin are inconclusive [44–46]. Moreover, in the upper small intestine a slower absorption, a more alkaline milieu, and the activity of intestinal HCE may facilitate hydrolysis to salicylate (Figure 3), thus lowering the bioavailability of aspirin from EC formulations. In agreement with this hypothesis, different groups have reported an incomplete serum TXB_2_ suppression in a fraction of subjects exposed to EC as compared to plain aspirin formulations [26, 47] (Table 2). Given that some studies administered to the same subjects different aspirin formulations and that subjects fully inhibited by plain aspirin were incompletely responsive to different EC formulations, a PD cause of an incomplete acetylation of platelet COX-1 associated with EC preparations can be ruled out. Thus, a different PK due to a variable absorption and bioavailability of EC formulations both presystemically and systemically, is likely. Moreover, whether a reduced bioavailability of EC formulation might lower the antithrombotic protection of aspirin especially at lower doses remains unproven. It may be conceivable that EC aspirin, especially when associated with conditions characterized by additional modifications of aspirin PK such as obesity, might affect the antithrombotic protection of the drug (Table 2).

 Obesity is known to affect the PK of several classes of drugs such as chemotherapies, psychotropic drugs, anaesthetics, opioids, and *β*-blockers [48, 49] due to a change in body composition, regional blood flow, modification of plasma proteins and/or tissue components, distribution volume, and kidney and hepatic clearance mechanisms. Moreover, in obese subjects, the activity of some CYP450s and phase II conjugation enzymes are increased, human adipose tissue upregulates HCE1 [50]. The PK of markedly lipophilic drugs is particularly affected by obesity as compared to less lipophilic ones [49]. Modification of PK mechanism(s) associated with obesity might contribute to a faster bioinactivation of aspirin inside and outside the liver. In fact, aspirin is a highly lipophilic molecule, and it is biotransformed by phase II conjugation (Figure 3). Consistently, an increased body weight has been associated with a lower biochemical responsiveness to aspirin, as assessed by TXB_2_ or platelet function assays [47, 51, 52] (Table 2), and with a possible lower clinical efficacy of low-dose aspirin [53] although the clinical impact of this phenomenon has never been formally tested in large trials.

Ageing is also associated with a modified, usually increased, drug responsiveness. The most important PK-related changes in old age include a decrease in the excretory capacity of the kidney and a decline in hepatic blood flow, hepatocyte mass, and consequent reduced hepatic drug bioinactivation [54]. In addition, comorbidities and polypharmacy are often interfering with drug response. A reduction of aspirin esterase and cholinesterase activity in frail elderly people has been reported [27, 55] (Table 3). For some drug classes, such as *β*-blockers and opioids, age-dependent PD changes have been described [55]. The elderly appear also more susceptible to drugs affecting hemostasis, a lower dose of warfarin is needed to reach the same therapeutic window in old versus younger patients, and it is associated with a higher bleeding tendency [55]. As far as aspirin is concerned, old people appear more sensitive (and responsive) to aspirin as compared to younger ones, measured as a degree of TXB_2_ inhibition, [51]. Moreover, gastrointestinal bleeding risk in aspirin-treated patients steeply increases in the older decades of life [56, 57]. From a PD point of view, serum TXB_2_ and platelet aggregation induced by arachidonic acid do not change with age in untreated healthy subjects [58, 59], possibly ruling out an age-related change in the aspirin's PD. Whether these biochemical data might affect the benefit/risk profile of aspirin in older subjects is unknown. The elderly generally have been underrepresented in clinical trials, creating many uncertainties and less optimal medical care. Ongoing trials are addressing the issue of aspirin risk/benefit profile in the elderly. The Japanese Primary Prevention Trial (JPPP) has completed in 2007 the enrolment of 14,460 high-risk patients aged between 60 and 85 yrs [60]. Patients are randomised to placebo or EC aspirin 100 mg/day, and the primary end point of this study is a composite of cardiovascular events. The Aspirin in Reducing Events in the Elderly (ASPREE) is also a primary prevention, placebo-controlled study, assessing the efficacy of daily 100 mg EC aspirin in reducing death from any cause, incident dementia or persistent physical disability in subjects aged ≥70 yrs, including also subjects without additional risk factors, aside from age [61].

 Some NSAIDs might create a transient status of reduced responsiveness to aspirin, due to a PD competition between the short-lived aspirin and some NSAIDs which have a relatively longer half-life, for the same Arg residue at the COX-1 active site [62]. Due to the over-the-counter access to these drugs and to the fact that NSAIDs are the most used drugs worldwide [63], it is hard to estimate the impact of this phenomenon in reducing efficacy and safety of aspirin's cardiovascular prevention. Recently, a large retrospective nationwide study showed that PPI use is associated with an increased risk in cardiovascular events in aspirin-treated patients [64]. It is unknown whether this effect is due to a PK drug-drug interaction, where PPIs, by increasing stomach pH, reduce aspirin's bioavailability, or there is an increased risk of cardiovascular events associated with the class of PPIs [65].

**Figure 4:**
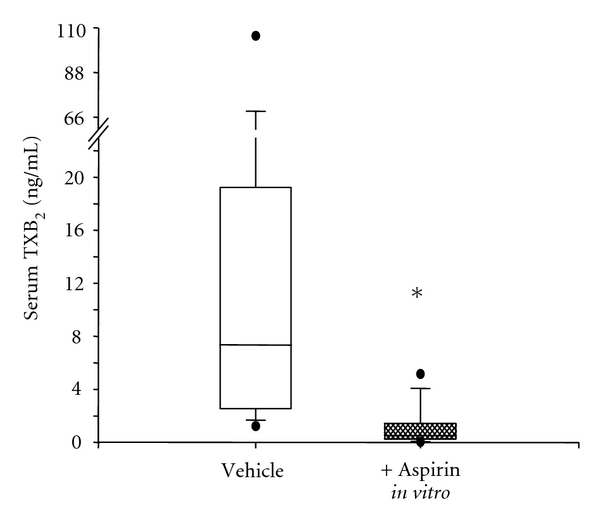
Serum TXB_2_ production from low-dose aspirin-treated patients with essential thrombocythemia *ex vivo* and after *in vitro* incubation of additional aspirin. Box-whisker plots of serum TXB_2_ values from 14 patients chronically treated with 100 mg/die aspirin, without (vehicle) and with *in vitro* incubation with 50 *μ*M aspirin. *P < 0.001. This figure is modified from [25].

Platelet turnover was hypothesized to influence the response to aspirin already more than 25 years ago. A pathophysiological condition of increased platelet generation is essential thrombocythemia (ET), a myeloproliferative neoplasm characterized by increased arterial thrombotic complications (myocardial infarction, stroke, or transient ischemic attack) [66], thus requiring antiplatelet treatment of prophylaxis. Previous studies from our group reported that aspirin-treated (100 mg once daily) patients affected by myeloproliferative neoplasms, and, more specifically, by ET, had a significant residual, uninhibited serum TXB_2_ [25, 67, 68]. While low-dose aspirin given once daily is capable of inhibiting by approximately 97% to 99% platelet TXA_2_ biosynthesis in healthy subjects [13, 17], the same aspirin regimen is unable to fully inhibit platelet TXA_2_ production in approximately 80% of ET patients [25]. The residual platelet COX (both COX-1 and -2) could be fully suppressed to levels comparable to controls by adding aspirin *in vitro* [25], thus indicating the presence of unacetylated platelet enzyme in at least a fraction of peripheral platelets (Figure 4) and ruling out changes in the drug target (platelet COX-1 and -2) which make it inaccessible or scarcely inhibitable by aspirin. Whether incomplete suppression of platelet COX activity in ET is due to disease-related changes in PK or PD of once-daily low-dose aspirin is currently unknown. Changes in aspirin PK in ET seem improbable due to the relatively young age of the patients, thus obesity, diabetes, other comorbidities, or polypharmacy are unlikely. On the other hand, due to a faster renewal of aspirin's drug target consequent to an enhanced platelet turnover, a disease-related PD change is both biologically and pharmacologically plausible. Thus, an accelerated platelet turnover might generate more unacetylated COX-1 and/or COX-2 during the 24-hour dosing interval, which would account for a partial recovery of TX-dependent platelet function, for example, the interval between two subsequent aspirin intakes.

Another disease associated with a lower-than-expected response to antiplatelet agents is type 2 diabetes mellitus (T2DM). Aspirin is currently recommended for T2DM independently of prior vascular complication [69, 70]. However, direct evidence for its clinical efficacy and safety in this setting is lacking [71, 72] or at best inconclusive [73, 74]. Once-daily administration of low-dose aspirin (75–100 mg) may be associated with incomplete inhibition of platelet COX-1 activity [75] and TX-dependent function [76, 77] in diabetics. PD or PK-related mechanisms might contribute to a reduced response in T2DM. Both in humans and animal models, diabetes is characterized by increased mean platelet volume, increased platelet mass, platelet turnover, and by morphological hallmarks of abnormal megakaryopoiesis [78–80]. Increased platelet turnover and abnormal megakaryopoiesis in T2DM might depend of diabetes itself or be secondary to an increased platelet consumption, likely at atherosclerotic lesions. However, previous reports on humans or animals suggest a primary disturbance of megakaryocytes in diabetes [80, 81]. Plasma aspirin esterases do not appear to be modified by T2DM [82]. Another PK-based mechanism reducing aspirin responsiveness in a fraction of patients might be obesity, often associated with T2DM, which may limit the antiplatelet effect of aspirin as shown in nondiabetic obese subjects. Another mechanism might be related to enhanced formation of lipid hydroperoxides limiting COX-isozyme acetylation by aspirin [83] in both megakaryocytes and circulating platelets.

## 4. How to Restrain Interindividual Variability in Response to Aspirin?

Lower responsiveness to aspirin can be a final, common phenotype, resulting from different PK- and/or PD-related mechanisms. Even though the final biochemical phenotype is a persistent, residual TXA_2_ generation from platelets not adequately inhibited by aspirin, understanding the underlying PK- or PD-related mechanisms is crucial to design strategies able to restore a normal response, restrain variability, and tailor the therapeutic intervention to the therapeutic need.

 Consistently with the hypothesis of a change in aspirin's PD on the basis of accelerated platelet turnover in T2DM, a substantial recovery of serum TXB_2_ between two subsequent low-dose aspirin dosing, for example, between 12 and 24 hours after aspirin intake, has been recently reported by our group [84] in a fraction of aspirin-treated T2DM patients. Similarly, in approx. 30% of stable coronary artery disease patients, a faster recovery of AA-dependent platelet function, measured 24 hours after dosing was associated with diabetes, smoking, and inflammatory biomarkers [85, 86]. Thus, some patients who are fully responsive to aspirin up to 6–12 hours after drug intake, ruling out PK modifications, show a substantial recovery of platelet's cyclooxygenase activity within the interval between dosing, for example, 24 hours. Thus, PD-related mechanisms, such as an increased platelet turnover or intraplatelet neosynthesis of COX-1 and/or -2 within 24 hours after dosing, have been hypothesized [84–87]. Consistently with this, hypothesis in a small group of healthy subjects, higher residual serum TXB_2_ while on aspirin was associated with the highest tertile of reticulated platelets, which are the youngest circulating platelets [88].

 To correct the reduced responsiveness to aspirin in ET or T2DM, small randomized studies tested different strategies, such as increased dose and/or more frequent drug administration. Almost simultaneously, P. Hjemdahal's and our groups have recently presented two studies in T2DM, with slightly different design and measurements (arachidonic-acid induced platelet aggregation or serum TXB_2_), both showing that a twice-daily low dose aspirin (75 or 100 mg) achieved an almost-complete and steady 24-hour platelet inhibition as compared to a single administration of a aspirin double dose (320 or 200 mg daily) [84, 87]. In both studies, reticulated platelets (RP) and/or mean platelet volumes (MPV) measured as indexes of increased platelet turnover were significantly correlated with a worse aspirin responsiveness. The effectiveness of a twice-daily dosing rather than of a double dose in reducing residual serum TXB_2_ or other platelet-related indexes is more consistent with a PD-, platelet turnover-based mechanism rather than with a lower bioavailability of aspirin. Thus, provided that low-dose aspirin PK is preserved and the drug target molecule is not changed (e.g., oxidatively damaged or structurally modified), then in conditions of accelerated megakaryopoiesis, a more frequent, rather than a higher doses might be needed and tested in large, randomized studies.

**Figure 5:**
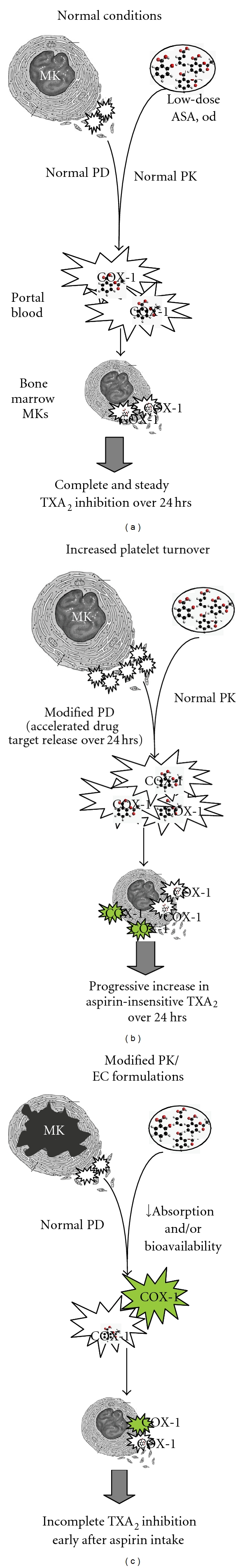
Models of variable responsiveness to low-dose aspirin given once daily. The figure depicts models of pharmacodynamic- (PD-) or pharmacokinetic- (PK-) related variable pharmacological response to aspirin, as measured by serum TXB_2_. Under normal conditions (left panel) aspirin inhibits peripheral platelets and bone-marrow megakaryocytes (MKs) resulting in a relatively steady inhibition of platelet COX activity over the 24 hour dosing interval. In case of increased platelet turnover (mid panel), the short-lived aspirin appears unable to acetylated new platelets which are released from MKs during the 24 hour dosing interval, thus resulting in a progressive increase in TXA_2_ generation between 12 and 24 hours after dosing. In case of variation in drug's PK (right panel), a reduced drug bioavailability in the portal and/or in the systemic circulation would lead to a suboptimal platelet TXA_2_ generation already at early time points (6–12 hours) after drug intake. Unacetylated platelets are represented in green. EC: enteric coated.

 On the other hand, if aspirin PK is modified, without any change in platelet turnover and drug target PD, as possibly the case in obese subjects for reduced bioavailability of the drug, then a dose increase might be sufficient to restore an aspirin-responsive phenotype. In a small study, doubling the once-daily aspirin dose in obese subject (from 75 EC to 150 plain formulation, mean body weight 120 kg) restored a nearly complete inhibition of serum TXB_2_ [47]. Thus, if a lower aspirin bioavailability is associated with obesity, without changes in aspirin PD, then one would expect that a small increase of the dose, within the low-dose range (<325 mg od), might be able to restore a normal pharmacological response to the drug. A model is depicted in Figure 5.

## 5. Conclusions

Aspirin within the low-dose range, administered mostly once daily, is the main antiplatelet drug for cardiovascular disease treatment, reducing on average by approximately 30% major cardiovascular events, especially MI, in high-risk patients. A substantial individual variability in biochemical drug responsiveness can be associated with specific physiologic (ageing), pathologic (ET, T2DM), or pharmacologic (NSAIDs, PPIs?) conditions due to transient (NSAID interaction and obesity) or stable (ET, T2DM, and ageing) changes in aspirin's PK and/or PD. Different aspirin regimens within the low-dose range, in small studies, have been able to restore a normal aspirin pharmacological responsiveness. Whether this variability can affect the clinical efficacy and safety of aspirin in cardiovascular prevention, especially in some primary prevention settings (T2DM), and the risk-benefit profile of alternative ways of giving aspirin are the next challenges in the aspirin scenario.

## Figures and Tables

**Table 1:** Main pharmacological features of drug resistance versus variability in drug response.

Resistance	Variability
(i) is usually *triggered by drug exposure*, *slowly reversible, *and* often *drug specific	(i) *not induced by and often independent of *the drug, can affect different drugs, and *does not revert* upon withdrawal

(ii) implies a *change of the drug target *making it inaccessible or no longer inhibitable	(ii) the drug target is not necessarily modified or inaccessible

(iii) detectable by *specific laboratory tests,* which *impact on the clinical decision* of *changing* drug	(iii) laboratory *tests are of little help* in therapeutic decisions if mechanism(s) are unknown (c*hange drug, increase dose, more frequent intake?*)

**Table 2:** Relative risk of incomplete (<95%) inhibition of TXB_2_ for a 10 kg increase in body weight.

Preparation	RR per 10 kg weight increase	95% CI
Asasantin b.i.d. (25 mg ASA plus 200 mg dipyridamole)	1.9	1.3–2.7
EC aspirin (75 mg)	2.2	1.7–3.0

Abbreviations: ASA: aspirin; EC: enteric coated. This table is modified from [26].

**Table 3:** Age-related plasma esterase activities.

Type of esterase	18–29 yrs	30–44 yrs	45–69 yrs	60–75 yrs	>75 yrs	>75 “fraile”
Ach esterase	2.78 ± 0.4	3.35 ± 0.1	3.1 ± 0.2	3.3 ± 0.2	3.13 ± 0.3	1.96* ± 0.6
Aspirin esterase	162 ± 27	161 ± 18	146 ± 11	147 ± 10	128 ± 22	64* ± 23

Correlation age-esterase activities: Ach *r* = −0.011, *P* = 0.9; ASA *r* = −0.25, *P* = 0.11; Ach: acetylcholine; yrs: years; *P < 0.005 versus nonfraile, old people. Data from [27, 28].
